# No significant short-term advantages of corticosteroids-augmented intra-articular injections of hyaluronic acid for knee osteoarthritis: A meta-analysis

**DOI:** 10.1007/s00132-025-04762-0

**Published:** 2026-01-09

**Authors:** Filippo Migliorini, Riccardo Giorgino, Francesco Oliva, Gennaro Pipino, Michael Kurt Memminger, Nicola Maffulli

**Affiliations:** 1 Department of Trauma and Reconstructive Surgery, University Hospital of Halle, Martin-Luther University Halle-Wittenberg, 06097 Halle (Saale), Germany; 2 Department of Orthopaedic and Trauma Surgery, Academic Hospital of Bolzano (SABES-ASDAA), 39100 Bolzano, Italy; 3 Department of Life Sciences, Health, and Health Professions, Link Campus University, 00165 Rome, Italy; 4 Residency Program in Orthopedics and Traumatology, University of Milan, Milan, Italy; 5 Faculty of Medicine, San Raffaele University, Rome, Italy; 6 Department of Orthopaedics, Villa Erbosa Hospital Gruppo San Donato, Bologna, Italy; 7 Department of Trauma and Orthopaedic Surgery, Faculty of Medicine and Psychology, University La Sapienza, 00185 Roma, Italy; 8 School of Pharmacy and Bioengineering, Keele University Faculty of Medicine, ST4 7QB Stoke on Trent, UK; 9 Centre for Sports and Exercise Medicine, Barts and the London School of Medicine and Dentistry, Mile End Hospital, Queen Mary University of London, E1 4DG London, UK

**Keywords:** Regenerative medicine, Orthopaedics, Viscosupplementation, Degeneration, Conservative management, Injections, Regenerative Medizin, Orthopädie, Viskosupplementation, Degeneration, Konservatives Management, Infiltrationen

## Abstract

**Introduction:**

The present meta-analysis investigated whether augmenting hyaluronic acid (HA) with corticosteroids (CCs) is more effective than HA in isolation in patients with knee osteoarthritis (OA). The outcomes of interest were the Western Ontario and McMaster Universities Osteoarthritis Index (WOMAC) scores.

**Methods:**

This study was conducted according to the 2020 PRISMA statement. In September 2025 the following databases were accessed: PubMed, Web of Science, Google Scholar and Embase. All head-to-head randomized controlled trials (RCTs) comparing intra-articular injections of HA in isolation versus HA augmented with CCs were accessed.

**Results:**

Data from 216 patients were collected and 74% (162 of 216) of the patients were women. The mean patient age was 57.7 ± 4.4 years and the mean body mass index (BMI) was 28.2 ± 3.0 kg/m^2^. No difference was found in WOMAC scores between 3–4 months of follow-up (*P* = 0.9); No difference was found in WOMAC scores between 4–6 months of follow-up (*P* = 0.5).

**Conclusion:**

The current level I of evidence suggests that intra-articular HA injections augmented with CCs do not appear to provide clinically relevant advantages in WOMAC outcomes within 3–6 months of follow-up.

**Supplementary Information:**

The online version of this article (https://doi.org/10.1007/s00132-025-04762-0) contains supplementary material, which is available to authorized users.

## Abstract (de)

**Einleitung:**

Die vorliegende Metaanalyse untersuchte, ob die Ergänzung von Hyaluronsäure (HA) mit Kortikosteroiden (CC) bei Patienten mit Gonarthrose wirksamer ist als HA allein. Die wichtigen Endpunkte waren die Scores des Western Ontario and McMaster Universities Osteoarthritis Index (WOMAC).

**Methoden:**

Diese Studie wurde gemäß der PRISMA-Erklärung 2020 durchgeführt. Im September 2025 wurden die folgenden Datenbanken durchsucht: PubMed, Web of Science, Google Scholar und Embase. Es wurden alle randomisiert-kontrollierten Studien (RCTs) herangezogen, in denen intraartikuläre Injektionen von HA allein mit HA in Kombination mit CC verglichen wurden.

**Ergebnisse:**

Es wurden Daten von 216 Patienten erhoben, von denen 74 % (162 von 216) weiblich waren. Das Durchschnittsalter der Patienten betrug 57,7 ± 4,4 Jahre und der durchschnittliche Body-Mass-Index (BMI) lag bei 28,2 ± 3,0 kg/m^2^. Es wurde kein Unterschied in den WOMAC-Scores zwischen 3 und 4 Monaten Follow-up festgestellt (p = 0,9); es wurde kein Unterschied in den WOMAC-Scores zwischen 4 und 6 Monaten Follow-up festgestellt (p = 0,5).

**Schlussfolgerung:**

Die derzeitige Evidenzklasse I deutet darauf hin, dass intraartikuläre HA-Injektionen in Kombination mit Kortikosteroiden innerhalb von 3 bis 6 Monaten nach dem Follow-up keine klinisch relevanten Vorteile im Vergleich zu den WOMAC-Scores bieten.

## Introduction

Osteoarthritis (OA) of the knee is a frequent degenerative articular disease that primarily affects older adults [1–4]. The estimated pooled global prevalence is 23% in patients aged 40 years and over [5, 6]. Morning stiffness, chronic knee pain and loss of function are the main symptoms of knee OA [7–12]. In advanced OA stages patients suffer from limitations and disabilities in activities and quality of daily living and often require joint replacement for end-stage conditions [13–16]. Given the lack of disease-modifying management for knee OA, the current therapeutic approach is based mainly on conservative and symptomatic interventions [6, 17]. The range of conservative treatment options includes intra-articular injections, which have proven to be safe and effective [18, 19]. Frequently used agents for intra-articular knee injection include hyaluronic acid (HA) and corticosteroids (CCs), both first-line therapies for patients with knee OA [2, 20].

The HA is an endogenous glycosaminoglycan contained in the synovial fluid of the joint and functions as a protective lubricant and flexible shock absorber [21–25]. In OA the composition of synovial fluid is altered [26, 27]. Both the molecular weight and the concentration of synovial HA decrease, potentially reducing its protective layer effect [28]. Intra-articular injection of HA can restore the beneficial functions of intact synovial fluid, thereby enhancing cartilage protection, preventing synovial inflammation and exerting direct and indirect analgesic effects [29, 30]. Although the current evidence indicates numerous benefits, including reduced chronic knee pain, increased mobility and function and delayed surgery for knee OA, the safety and superiority of intra-articular HA injections over other agents remains the subject of debate [31–35].

In 1958 Miller et al. performed the first clinical trial of intra-articular CCs in knee OA [36]. Currently, HA is a well-accepted intra-articular treatment for knee joint OA as it has pronounced anti-inflammatory and immunosuppressive effects and increases the relative viscosity and concentration of endogenous HA in arthritic knees [37, 38]; however, the duration of efficacy of intra-articular CCs injection remains a subject of debate [39, 40].

An approach to improving and prolonging the effectiveness of the described treatment methods is to combine the individual active ingredients to produce a synergistic effect [41]. Despite extensive literature on the promising effects of the two active ingredients, there is a lack of rigorous scientific studies that combine HA and CCs in intra-articular knee joint injections. Therefore, the present meta-analysis investigated whether augmenting HA with CCs is more effective than HA in isolation in patients with knee OA. The outcome of interest was to examine whether HA augmented with CCs shows a comparable or different Western Ontario McMaster Universities Osteoarthritis Index (WOMAC) score compared to HA injection in isolation.

## Methods

### Eligibility criteria

All the head-to-head randomized controlled trials (RCTs) comparing intra-articular injections of HA in isolation versus HA augmented with CCs were accessed. Only studies published in peer-reviewed journals were considered. Given the author’s language capabilities, articles published in English, German, Italian, French and Spanish were eligible. Only studies with level I evidence, according to the Oxford Centre of Evidence-Based Medicine [42] were considered. Only studies which clearly stated that injections have been performed in patients with knee OA, irrespective of the severity of OA, were considered. Studies which compared HA with other biologically active non-HA treatment (e.g., platelet-rich plasma, PRP, CCs) were not included. Studies which evaluated intra-articular HA injections augmented with other biologically active compounds were not included. Studies that were regarded as comparators of other noninfiltrative therapies were not eligible.

### Search strategy

This study was conducted according to the Preferred Reporting Items for Systematic Reviews and Meta-Analyses: the 2020 PRISMA statement [43]. The PICOD algorithm was preliminarily established:

- P (Problem): knee OA;
- I (Intervention): intra-articular HA injections;
- C (Comparison): HA v.s HA augmented with CCs;
- O (Outcomes): WOMAC;
- D (Design): RCT.

In September 2025 the following databases were accessed: PubMed, Web of Science, and Embase. No time constraint was set for the search. The Medical Subject Headings (MeSH) used for the database search are reported in the Appendix. No additional filters were used in the database search.

### Selection and data collection

The database search was performed by two authors (RG & GP). All resulting titles were screened per hand and, if suitable, the abstract was accessed. The full text of the abstracts which matched the topic was accessed. If the full text was unavailable, the article was not considered for inclusion. A cross-reference of the full-text article bibliographies was also performed for inclusion. Disagreements were debated and mutually solved by the authors. In the case of further disagreement a third senior author (NM) made the final decision.

### Data items

Two authors (RG & GP) performed data extraction. The following baseline data were extracted: author, year of publication and journal, length of follow-up, number of patients, and the mean age and BMI. Data on the WOMAC [44] were retrieved at baseline and at the last follow-up. Data were extracted in Microsoft Office Excel version 16.72 (Microsoft Corporation, Redmond, WA, USA). Concerning the WOMAC score, 24 health-specific items covering pain (5 items), stiffness (2 items) and function (17 items) were assessed. The subscale scores for pain, stiffness and function are summed to produce the total score; however, WOMAC subscale data were not consistently reported across the included studies and only the total WOMAC score could be extracted in a sufficiently comparable format for the meta-analysis. Scores range from 0 (least pain) to 20 (highest pain) for pain, 0 (least stiffness) to 8 (greatest stiffness) for stiffness, 0 (best function) to 68 (worst function) for function and 0 (best health) to 96 (worst health) for the total score. Higher scores represent worse pain, stiffness, and functional limitations.

### Methodological quality assessment and quality of the recommendations

The risk of bias was assessed in accordance with the Cochrane Handbook for Systematic Reviews of Interventions [45]. Two reviewers (RG & GP) evaluated the risk of bias in the extracted studies. Disagreements were solved by a third senior author (NM). The RCTs were evaluated using the revised Risk of Bias assessment tool (RoB2) [46, 47] of the Cochrane tool to assess the risk of bias in randomized trials (RoB). The following endpoints were evaluated: bias arising from the randomization process, bias based on the deviations from intended interventions, bias because of missing outcome data, bias in the measurement of the outcome and bias in the selection of the reported result.

### Synthesis methods

The main author (FM) performed the statistical analyses following the recommendations of the Cochrane Handbook for Systematic Reviews of Interventions [48]. The IBM SPSS Statistics version 25 (IBM Corporation, Armonk, NY, USA) was used to perform descriptive statistics. The arithmetic mean and standard deviation were used. For the meta-analyses, Review Manager 5.3 (The Nordic Cochrane Collaboration, Copenhagen, Denmark) was used. The inverse-variance method with the mean difference (MD) effect measure was used for continuous data. The confidence interval (CI) was set at 95% in all the comparisons. Heterogeneity was assessed using the Higgins I^2^ statistic and the χ^2^ test. If P_χ2_ > 0.05, no statistically significant heterogeneity was found. If P_χ2_ < 0.05, heterogeneity was evaluated using the Higgins I^2^ statistic. Suppose the Higgins‑I^2^ test > 70%, high heterogeneity was found. A fixed effect model was set as the default. If high heterogeneity was detected, a random model effect was used. As none of the comparisons exceeded the predefined threshold for substantial heterogeneity (I^2^ > 70%), all meta-analyses were ultimately conducted using fixed-effects models and *P*-values < 0.05 were considered statistically significant.

## Results

### Study selection

**Fig. 1:**
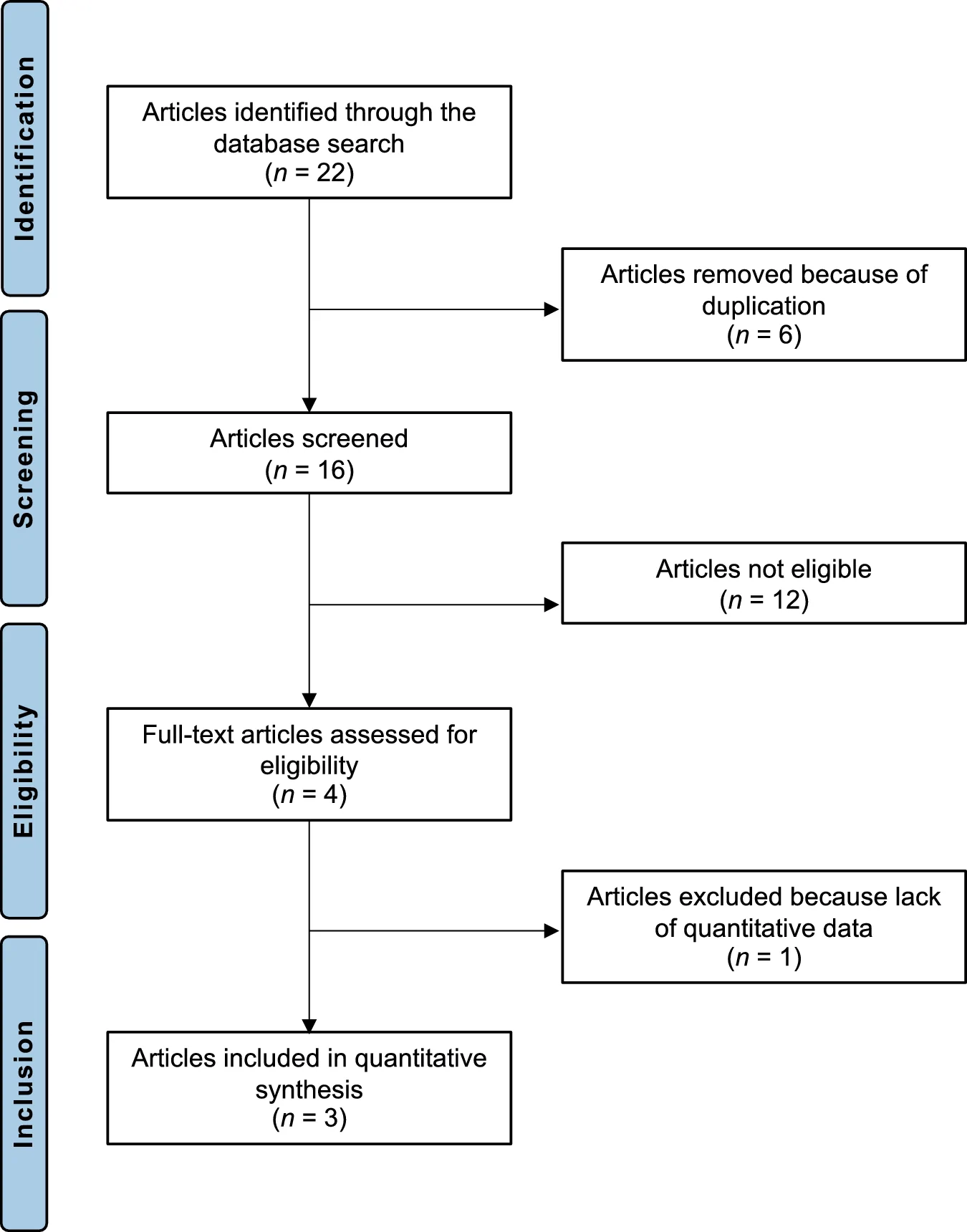
PRISMA flow chart of the literature search

The systematic literature search identified 22 studies of which 6 were identified as duplicates and were therefore excluded. Screening the abstracts of the remaining 16 investigations for eligibility excluded 12 additional articles. The detailed reasons that led to exclusion were the following: study type and design (*N* = 2), comparing HA with other biologically active non-HA treatments (*N* = 3), evaluating intra-articular HA injections augmented with other biologically active compounds (*N* = 4), considering other noninfiltrative therapies as comparators (*N* = 1), and language limitations (*N* = 2). A further study lacked quantitative data on outcomes of interest and was therefore not considered. In conclusion, three RCTs were selected for inclusion in the present investigation. The results of the literature search are shown in Fig. 1.

### Methodological quality assessment

**Fig. 2:**
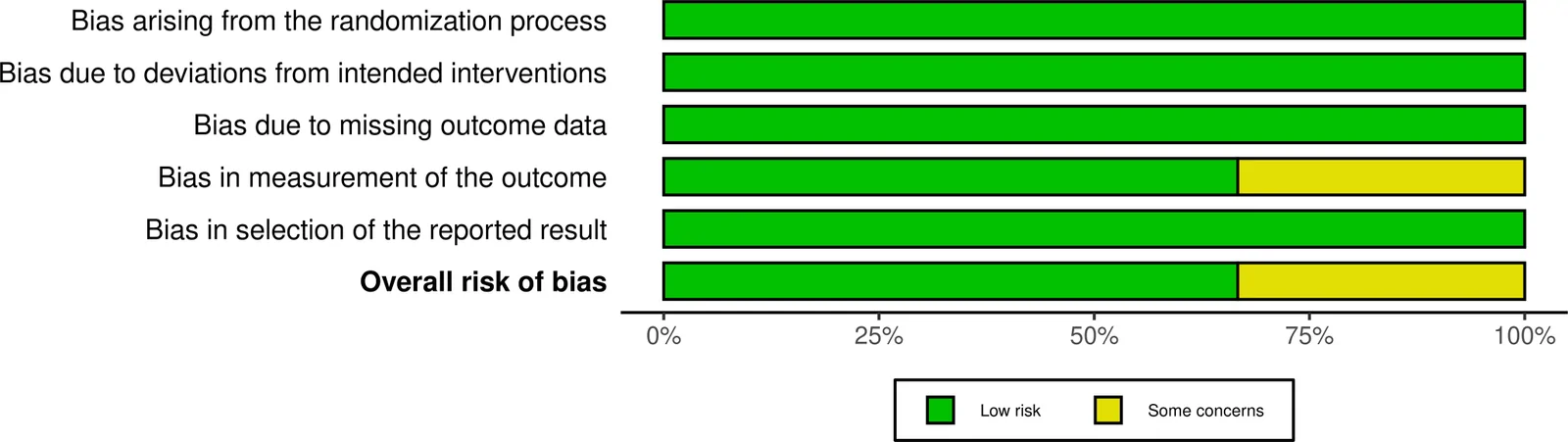
Cochrane risk of bias tool. (RoB2)

The risk of bias for the RCTs included in the present meta-analysis was assessed using the revised risk of bias assessment tool (RoB2). The assessment identified no concerns during the randomization process in all of the studies addressing the comparability of the groups studied at baseline, which resulted in a low risk of bias in this domain. The risk of bias based on the deviations from the intended intervention, missing outcome data and selection of the reported result was low in all the RCTs evaluated. Only measuring the outcome resulted in concerns, given the lack of blinding processes in two trials. In summary, the risk of bias graph indicates a high methodological quality of the RCTs included in the present investigation (Fig. 2).

### Study characteristics and results of individual studies

Data from 216 patients were collected and 74% (162 of 216) of the patients were women. The mean age of the patients was 57.7 ± 4.4 years and the mean BMI was 28.2 ± 3.0 kg/m^2^. The characteristics and demographics of the included studies are shown in Table 1.

**Table 1:** Generalities and demographics of the included studies

Author, year	Journal	Intervention	Patients (*n*)	Mean age (years)	Women (*%*)	Mean BMI (*kg/m*^*2*^)
De Campos et al. [49]	*Clin Orthop Relat Res*	HA	52	61.0	75	30.0
		HA and CCs	52	65.0	77	29.0
Huang et al. [50]	*Orthopade*	HA	40	54.8	84	24.5
		HA and CCs	40	54.3	83	24.6
Maia et al. [51]	*Clinics (Sao Paulo)*	HA	16	56.6	63	31.9
		HA and CCs	16	54.5	63	29.0

*BMI* body mass index; *CCs* corticosteroids; *HA* hyaluronic acid

### Baseline of the groups

An overview of the data of each group is reported in Table 2.

**Table 2:** Baseline comparability

Endpoint	*P*
Mean age (*year*)	0.9
Women (*%*)	0.9
Mean BMI (kg/m^2^)	1.0
Mean WOMAC	0.7

*BMI* body mass index; *WOMAC* Western Ontario and McMaster Universities Osteoarthritis Index

### Results syntheses

**Fig. 3:**
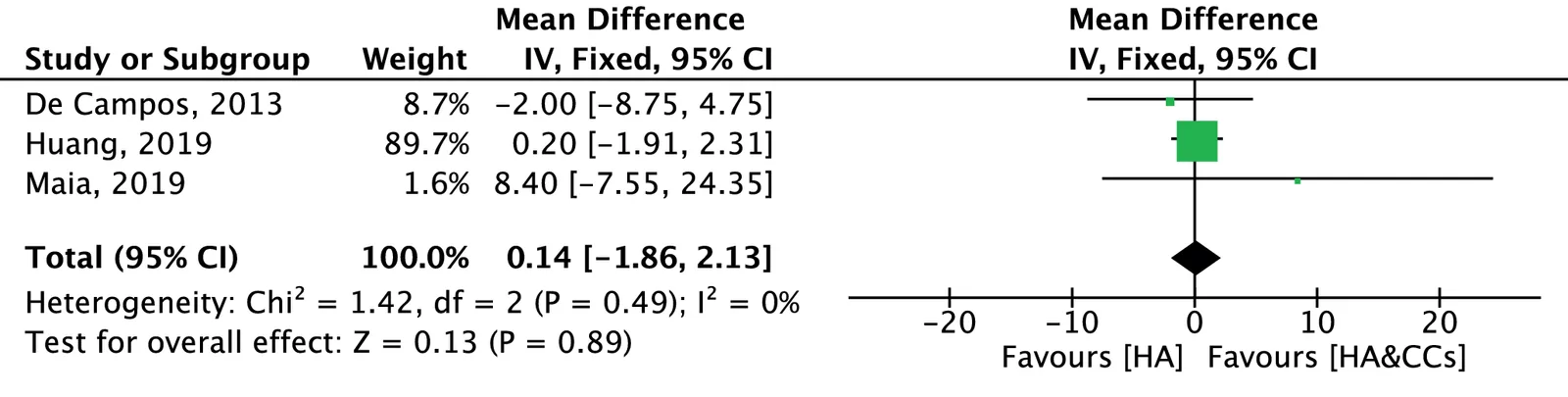
Results of the meta-analyses at the time point 3–4 months (*CI* confidence interval)

No difference in WOMAC was observed at 3–4 months of follow-up (MD 0.14; 95% CI −1.86 to 2.13; *P* = 0.9). Heterogeneity was negligible (I^2^ = 0%) (Fig. 3).

**Fig. 4:**
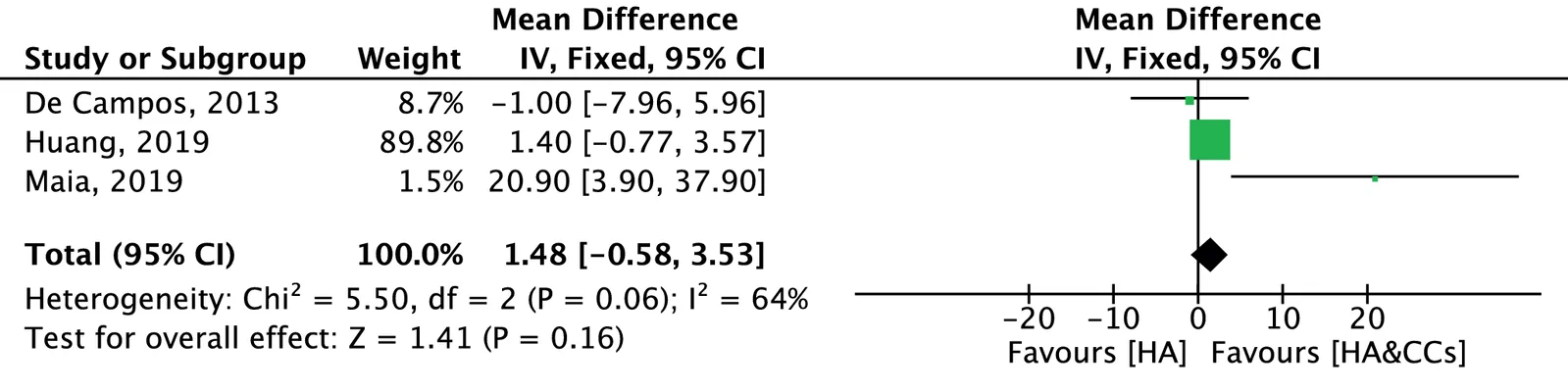
Results of the meta-analyses at the time point 4–6 months (*CI* confidence interval)

No difference in WOMAC was observed at 4–6 months of follow-up (MD 1.48; 95% CI −0.58 to 5.53; *P* = 0.5). Heterogeneity was moderate (I^2^ = 64%) (Fig. 4).

## Discussion

According to the main findings of the present meta-analysis, the current level I evidence indicates that augmenting CCs with HA intra-articular injections is not associated with improved WOMAC score efficacy within 3–6 months of follow-up.

De Campos et al. [49] included 104 patients evenly divided between those treated with HA alone and those treated with HA combined with CCs. The results showed an improvement in symptom and functional scores in the first week in the group that received added triamcinolone, but no significant difference in subsequent months [49]. There was no increased likelihood of adverse effects [49]. Although triamcinolone improved efficacy in the short term, it did not improve long-term outcomes, suggesting that combined triamcinolone and HA only temporarily alleviates symptoms, raising doubts about its long-term relevance [49]. Huang et al. [50] compared 80 patients treated with HA alone and those with the combination of HA and CCs. The results at 3 months of follow-up did not reveal significant differences in WOMAC scores [50]. Furthermore, no significant differences were confirmed at 4–6 months [50]. Finally, following a similar pattern of results, a prospective randomized controlled trial by Maia et al. [51] evaluated the effects of intra-articular injection of HA and dexamethasone, administered in isolation and in combination, in 32 patients. The use of HA alone significantly improved the total WOMAC score and pain, stiffness, and function subscores up to 6 months post-injection [51]. Additionally, HA alone improved the muscle strength of knee extensors and flexors during the same period [51]. No treatment affected proprioception [51]. These findings suggest that HA positively affects arthrogenic inhibition of the quadriceps [51]; however, combining with dexamethasone did not yield further significant improvements [51].

These findings consistently indicate that CC augmentation does not improve mid-term functional or symptomatic outcomes following HA injection. The evaluation was conducted only at 3–4 and 4–6 months, potentially overlooking immediate variations or long-term differences. The progressive nature of OA may require assessments over longer periods to detect potential benefits of adding CCs. Importantly, our analysis was restricted to mid-term outcomes (3–6 months) and therefore cannot exclude the possibility of early short-term benefits associated with CC augmentation, which typically occur within the first weeks following injection.

The efficacy of HA and CCs remains controversial in the literature. While HA is well-established for its efficacy in knee OA [52, 53], the intra-articular use of CCs remains debated, given their non-modifying effect on the condition and their short duration of analgesic efficacy [54]. Despite this, its use for OA is recommended [55]. The results of our study, which evaluated a relatively short follow-up period, are inconsistent with those of other studies in the literature. In the past, the combination of these two compounds had proven efficacy in improving pain and function in the first weeks [56, 57]; however, these results are limited to the considered follow-up period and further research is needed to assess long-term effects and determine whether any subgroups of patients might benefit from this therapeutic combination.

The present study has several limitations that must be considered when interpreting the results. First, the main limitation of this meta-analysis is the use of the total WOMAC score as the sole outcome measure. This approach may not capture specific variations in pain subscales and other aspects, such as stiffness and overall joint function, limiting the interpretability of the results in clinical terms; however, a subscale meta-analysis was not feasible because two of the three included RCTs did not report WOMAC subscale data in a sufficiently detailed and consistent format for extraction. As a consequence, the analysis relied exclusively on total WOMAC scores, which may mask domain-specific short-term effects, particularly regarding the early analgesic effect typically associated with CCs. Future trials should systematically report all WOMAC subscales in a standardized manner.

Another significant limitation concerns the assessment of results at only 3–4 and 4–6 months of follow-up. This approach may not capture immediate variations in treatment response or account for the long-term effectiveness of adding CCs to HA injections. Moreover, the studies included in the meta-analysis used HA molecules with different molecular weights. This diversity could influence the viscosity and persistence of the therapeutic effect of HA, adding variability that might impact overall results. Additionally, a clear standardization of indications for HA and CCs appears to be lacking. This variability in indications could influence treatment response and introduce a confounding factor into the results. Another limitation is the diversity of treatment protocols, including session frequency and the number of substances used. Another relevant limitation of the present meta-analysis concerns the heterogeneity of the hyaluronic acid formulations used across the included RCTs. The studies differed in molecular weight, viscosity, and HA preparation characteristics, and these differences were not consistently or sufficiently reported to permit reliable stratification. Performing a subgroup or sensitivity analysis based on HA molecular weight was not methodologically feasible and would have severely underpowered the comparisons. As a consequence, the influence of HA characteristics on treatment effect could not be adequately assessed, representing a potential confounder that should be considered when interpreting the results. Moreover, not all included RCTs reported OA severity, injection protocol details, or HA molecular weight, preventing standardized inclusion of these variables in Table 1. Finally, the high BMI among the included patients is a relevant confounding factor to consider as a limitation. Obesity influences the course of OA and treatment response. The absence of a BMI-based subdivision could limit the understanding of differences in treatment response among patient subgroups. Other factors, such as age, gender, severity of OA and comorbidities can also vary significantly, influencing treatment response. Another methodological limitation is that the review protocol was not prospectively registered in an online registry such as PROSPERO. The absence of protocol registration may reduce transparency and introduce a theoretical risk of selective reporting.

In summary, the findings of the present meta-analysis should be interpreted with caution, as they are based on only three randomized controlled trials with short-term assessments (3–6 months). Within this limited evidence base, adding CCs to intra-articular HA injections does not appear to confer clinically meaningful benefits based on the WOMAC score; however, early benefits in the first weeks cannot be excluded and larger methodologically robust trials with longer follow-up are required to validate and extend these preliminary observations. Future randomized controlled trials should also employ more homogeneous HA formulations or at least provide detailed and standardized reporting of HA characteristics to clarify whether specific HA profiles may influence the effect of CCs.

## Conclusion

The current level I of evidence suggests that augmenting CCs to HA intra-articular injections does not appear to improve WOMAC outcomes in the mid-term. These findings should be considered preliminary and larger high-quality trials with longer follow-up are necessary to confirm or refute the potential benefits of this therapeutic combination.

## Supplementary Information

ESM1: Supplementary material 1

## Data Availability

The datasets generated and/or analyzed during the current study are available throughout the manuscript.

## Abbreviations

CCs: corticosteroids
CI: confidence interval
HA: hyaluronic acid
MD: mean difference
OA: osteoarthritis
PRP: Platelet-rich plasma
RCTs: randomized controlled trials
WOMAC: Western Ontario and McMaster Universities Osteoarthritis Index
