# Supplementary material for: No significant short-term advantages of corticosteroids-augmented intra-articular injections of hyaluronic acid for knee osteoarthritis: A meta-analysis
Source: Orthopadie (Heidelb). 2026 Jan 9;55(10):825–32. doi: 10.1007/s00132-025-04762-0 (PMC13601200; doi:10.1007/s00132-025-04762-0)
Supplement: Supplementary file 1 [file 132_2025_4762_MOESM1_ESM.pdf]

Systematic literature search for:

Corticosteroids augmented intra-articular infiltrations of hyaluronic acid for knee osteoarthritis: a meta-analysis

Concept 1: Knee osteoarthritis

Keywords:
knee OA
knee osteoarthritis

Mesh:
"Osteoarthritis, Knee"[Mesh]

Concept 2: Treatment

Keywords:
hyaluronic acid infiltrations
HA infiltrations
Corticosteroids (CCs)

Mesh:
"Hyaluronic Acid"[Mesh]

Concept 3: Interest of the outcome

PROMs

Keywords:
Western Ontario and McMaster Universities Osteoarthritis (WOMAC)

Mesh:
"Patient Outcome Assessment"[Mesh]
"Patient Reported Outcome Measures"[Mesh]

Concept 1:
"Osteoarthritis, Knee"[Mesh] OR knee osteoarthritis OR knee OA

AND

Concept 2:
"Hyaluronic Acid"[Mesh] OR hyaluronic acid infiltrations OR HA infiltrations

AND

Corticosteroids OR CCs OR corticosteroid therapy

AND

Concept 3:

"Patient Outcome Assessment"[Mesh] OR "Patient Reported Outcome Measures"[Mesh] OR PROM OR Western Ontario and McMaster Universities Osteoarthritis (WOMAC)

NOT

Hip

Summary of the Search

(((("Osteoarthritis, Knee"[Mesh] OR knee osteoarthritis OR knee OA) AND ("Hyaluronic Acid"[Mesh] OR hyaluronic acid infiltrations OR HA infiltrations)) AND (Corticosteroids OR CCs OR corticosteroid therapy)) AND ("Patient Outcome Assessment"[Mesh] OR "Patient Reported Outcome Measures"[Mesh] OR PROM OR Western Ontario and McMaster Universities Osteoarthritis (WOMAC))) NOT (Hip)
